# Neural substrates of figurative language during natural speech perception: an fMRI study

**DOI:** 10.3389/fnbeh.2013.00121

**Published:** 2013-09-19

**Authors:** Arne Nagels, Christina Kauschke, Judith Schrauf, Carin Whitney, Benjamin Straube, Tilo Kircher

**Affiliations:** ^1^Department of Psychiatry and Psychotherapy, Philipps-University MarburgMarburg, Germany; ^2^Department of Germanic Linguistics, Philipps-University MarburgMarburg, Germany; ^3^Department of Psychology and York Neuroimaging Centre, University of YorkYork, UK

**Keywords:** figurative speech, simile, abstractness, inferior frontal gyrus, fMRI

## Abstract

Many figurative expressions are fully conventionalized in everyday speech. Regarding the neural basis of figurative language processing, research has predominantly focused on metaphoric expressions in minimal semantic context. It remains unclear in how far metaphoric expressions during continuous text comprehension activate similar neural networks as isolated metaphors. We therefore investigated the processing of similes (figurative language, e.g., “He smokes like a chimney!”) occurring in a short story. Sixteen healthy, male, native German speakers listened to similes that came about naturally in a short story, while blood-oxygenation-level-dependent (BOLD) responses were measured with functional magnetic resonance imaging (fMRI). For the event-related analysis, similes were contrasted with non-figurative control sentences (CS). The stimuli differed with respect to figurativeness, while they were matched for frequency of words, number of syllables, plausibility, and comprehensibility. Similes contrasted with CS resulted in enhanced BOLD responses in the left inferior (IFG) and adjacent middle frontal gyrus. Concrete CS as compared to similes activated the bilateral middle temporal gyri as well as the right precuneus and the left middle frontal gyrus (LMFG). Activation of the left IFG for similes in a short story is consistent with results on single sentence metaphor processing. The findings strengthen the importance of the left inferior frontal region in the processing of abstract figurative speech during continuous, ecologically-valid speech comprehension; the processing of concrete semantic contents goes along with a down-regulation of bilateral temporal regions.

## Introduction

Figurative expressions are an established part of everyday speech and are often fully conventionalized. For example, parts of the human body are used in a multitude of figurative expression (head of department, eye of needle, arm of tree, etc.). Thus, figurative speech use goes far beyond the concept of a mere stylistic device and can be seen as an integral part of day-to-day communication. So far, it remains unclear in how far the comprehension of figurative speech draws on additional resources when presented in a naturally-evolving continuous and coherent story. Increases in activation of the left inferior frontal gyrus (IFG) have previously been reported for isolated sentences that contained figurative as opposed to non-figurative elements (Rapp et al., 2004, 2007; Kircher et al., 2007). However, prior context and repeated exposure to figurative speech—as it appears in more natural environments—can have an impact on how we process figures of speech such as similes. Context and conventionalization may facilitate comprehension (for idiom comprehension see Gibbs, 1992) and therefore neural activation of contextualized similes might not differ from non-simile sentences.

The current study focuses on the processing of figurative speech in form of similes such as “The sun is like the eye of heaven” in a natural context without constraining instructions or predetermined cognitive tasks, e.g., decision tasks; asking the participants to press a button, when either abstract or concrete content was presented.

A simile, such as “The sun is like the eye of heaven,” can be divided into three different components: (1) the explained element (“the sun”), (2) the explaining element (“the eye of heaven”), (3) and the term of comparison (TOC; “is like”), which connects the two elements in the simile (Leech, 1969). For successful comprehension, the listener needs to refer back to the explained element and identify similarities or common features with the corresponding explaining element (e.g., “He smokes like a chimney!” stresses that someone smokes heavily). The aspect that the two elements have in common is the so-called tertium comparationis, i.e., the third domain involved in a comparison. According to prevailing theories, similes are strongly linked to metaphors, which can be regarded as similes without the TOC (i.e., elliptical similes; cf. Aristotle).

When metaphors are presented, the listener is confronted with a semantic conflict between the explained and explaining element, which needs to be resolved. The initiator of a figurative utterance selects, emphasizes, suppresses, and organizes features of the explained element by applying characteristics of the explaining elements. Thus, a “mental linkage” between the explained element and the corresponding explaining element is required which goes beyond the usual semantic or word-by-word analysis (Rapp et al., 2004). The TOC (“like”) in similes may facilitate this linking process, since it explicitly points to the comparative nature of the utterance.

Usually, similes or metaphors do not stand alone but are integrated into a speech or text. This context can alter the ease by which meaning is integrated, including processing of figures of speech (Gibbs, 1992). Prior context facilitates the comprehension of idioms when it is consistent with the specific entailments of the idiom (i.e., their conceptual representation): though there might be different ways of expressing anger in an idiomatic way (e.g., “bite your head-off” as opposed to “blow your stack”), it is easier to comprehend idioms whose specific conceptual representation has been primed by previous information e.g., describing anger in a way that refers to anger as “animal behavior” (Nayak and Gibbs, 1990). A coherent and evolving story can provide such information, and participants should therefore understand figurative expressions that are embedded in a story easier than isolated sentences or sentence pairs with little detail (e.g., Rapp et al., 2007; Schmidt and Seger, 2009). Behavioral studies of other complex semantic operations, such as ambiguity processing, have also shown that cognitive resources can be saved when the target item is embedded in semantically coherent context as opposed to isolated or neutral environments [for a review see Simpson (1994)]. Also, the amount of prior, coherent information can facilitate inference making and improve comprehension of non-figurative material (Zwaan and Radvansky, 1998).

A number of functional Magnetic Resonance Imaging (fMRI) studies have investigated the neural correlates of figurative speech mostly in the form of metaphoric sentences (Rapp et al., 2004, 2007; Eviatar and Just, 2006; Kircher et al., 2007; Mashal et al., 2007, 2009; Shibata et al., 2007; Stringaris et al., 2007; Schmidt and Seger, 2009; Desai et al., 2011; Diaz and Hogstrom, 2011; Diaz et al., 2011), and for similes (Shibata et al., 2012). The brain response to similes in a story context, however, remains unexplored, thus far. The results of these previous studies support the involvement of the left lateral prefrontal cortex [for a critical review on the neural basis of metaphor processing see Schmidt et al. (2010)], as a correlate for increased cognitive demand during the comprehension of figurative language. In particular, Rapp et al. (2004) for the first time reported enhanced cortical activation in the left IFG [Brodmann area (BA) 45/47] for metaphor reading as compared to literal sentences. The left IFG is an integral component of the semantic processing network and has been related to executive aspects of meaning retrieval, such as semantic search, retrieval, selection, and integration (e.g., Thompson-Schill et al., 1997; Wagner et al., 2001; Noppeney et al., 2004; Badre et al., 2005; Badre and Wagner, 2007; Bedny et al., 2008; Binder et al., 2009). Moreover, difficult metaphors in comparison to easy metaphors such as “Political success is a house of cards” vs. “Books are treasure chests of information” (Schmidt and Seger, 2009) as well as anomalous metaphors [“Their (financial) capital has a lot of rhythm' (Ahrens et al., 2007)] were found to selectively activate the left IFG. Similarly, conventional metaphors as compared to novel metaphors were found to engage the left IFG, whereas novel metaphors activated the left middle frontal gyrus (LMFG) during a reading paradigm (Mashal et al., 2007). The graded response in prefrontal cortex, particularly the left IFG, suggests that activation correlates with the level of cognitive-semantic resources required for successful performance (e.g., integration effort). This is in accordance with previous semantic retrieval studies of graded difficulty, which showed enhanced left IFG response during tasks of high vs. low executive-semantic demands (Thompson-Schill et al., 1997; Roskies et al., 2001; Wagner et al., 2001; Badre et al., 2005; Snyder et al., 2007; Zempleni et al., 2007; Kuperberg et al., 2008; Nagel et al., 2008; Ruff et al., 2008; Snijders et al., 2009; Whitney et al., 2009a,b). A recent study found activations in the head of the caudate presenting metaphors in a context (Uchiyama et al., 2012). However, the majority of past studies on figurative language comprehension utilized highly controlled sentence reading paradigms with minimal prior information (Rapp et al., 2004, 2007; Eviatar and Just, 2006; Stringaris et al., 2006, 2007; Mashal et al., 2007; Yang et al., 2009). The observed left IFG activations for metaphors might partly reflect executive-semantic processes that are related to the lack of sufficient contextual priming, as it would occur in naturally evolving texts.

The aim of the current study was therefore to analyze the neural responses to simile processing and determine the involvement of the left IFG when similes were presented within a natural, unconstrained short story context. Naturalistic stimuli were successfully investigated in the context of different experimental fMRI paradigms (Hasson et al., 2004; Skipper et al., 2007; Domahs et al., 2012). However, the neural correlates of understanding similes in a short story have not been investigated, so far. We hypothesized left IFG activation during processing of sentences containing similes (e.g., he jumps like a gazelle) vs. literal sentences of comparable frequency, plausibility, comprehensibility, and length. In addition, we expected significant correlations between unfamiliar as well as highly abstract similes and enhanced blood-oxygenation-level-dependent (BOLD) responses in the left frontal region.

## Materials and methods

### Participants

Initially, 19 male subjects took part in the fMRI study. Due to head movement, data of three subjects had to be discarded from further analysis. For the remaining 16 participants movement was minimal as the maximum change in translation and rotation for each participant was less than one voxel size (i.e., 3.5 mm) and less than 1°, respectively. All 16 participants (mean age = 27.00 years, *SD* = 6.65; mean years of education = 14.50 years, *SD* = 1.67) were native speakers of German, right-handed according to the Edinburgh Inventory of Handedness (Oldfield, 1971) and showed average or above average verbal IQ as assessed by the German MWT-B multiple choice vocabulary test (Lehrl et al., 1995; mean estimated verbal IQ = 120.06, *SD* = 17.16). Subjects with recent substance use or general MRI incompatibility (e.g., metal implants) were excluded. All subjects gave informed consent and were paid 20 Euros for participation in the study. The local ethics committee at RWTH Aachen University approved the study.

### Stimuli

A slightly modified version of the short story “Der Kuli Kimgun” by Dauthendey (1930) was chosen for this study as in Whitney et al. (2009b). Low-frequent or foreign words were substituted by more familiar or high-frequent words. The final version included a total of 3581 words.

In general, short stories are well-structured narratives restricted to a few protagonists and basic narrative events which, together, are ascribed to a single, central conflict. Our story was written from an omniscient perspective, leaving out elaborate descriptions of the character's emotional or mental states. The sequence of events occurs chronologically, which allows the listener to build up a temporally continuous mental representation of the story. The short story chosen for the current fMRI study contained figurative descriptions of events and situations.

For auditory presentation during fMRI, the story was professionally recorded and spoken in a natural way by a trained, male speech therapist. The duration of the story was 23:32 min.

### Procedure

The story was presented via MRI compatible headphones in two successive runs lasting 14:32 and 9:00 min, respectively. Participants were instructed to close their eyes and listen to the story carefully. To make sure that subjects attended to the content, they were informed at the beginning of the experiment about a short interview after the MRI session about the content of the story. Hereby, 10 questions regarding critical episodes of the short story had to be answered.

### Figurative and control sentences

First, 32 similes as well as 50 randomly chosen control sentences (CS) were extracted from the short story independently by two linguists (Christina Kauschke and Judith Schrauf). In a behavioral test, the isolated similes and CS were rated by 20 volunteers, who did not take part in the fMRI study, according to the dimensions “plausibility,” “comprehensibility,” and “figurativeness.” For this purpose, an analogue scale from 1 to 7 was used. Regarding the plausibility rating the instruction was as follows: “Please rate the subsequent sentences according to their plausibility. Very plausible sentences describe ordinary events being easy to follow.” Comprehensibility was rated according to the instruction: “Please rate the subsequent sentences according to their comprehensibility. Very comprehensible sentences are those where the meaning can be understood easily and within a very short period of time.” Regarding the dimension figurativeness, the rating instruction was formulated: “Sentences can be distinguished with regard to their properties to evoke inner pictures from the content being conveyed. The figurative content can be perceived faster and be grasped more easily in some sentences. Please rate the degree of figurativeness in the following sentences.”

For the imaging analysis, 30 similes and 30 CS were chosen so that no significant differences were found between the similes and the CS according to the dimensions of plausibility [*F*_(1.58)_ = 2.68, *p* = 0.108], comprehensibility [*F*_(1.58)_ = 493, *p* = 0.49], and figurativeness [*F*_(1.58)_ = 1.76, *p* = 0.19]. The similes were found to be rather unfamiliar [mean = 2.91 (*SD* = 2.14)] and abstract [mean = 4.86 (*SD* = 2.29)]. All similes and CS were matched according to word frequency as well as to the number of syllables. Similes in comparison to CS revealed no significant differences with regard to their individual length in the story.

### fMRI data acquisition

All scanning was performed on a 1.5 T scanner (Gyroscan Intera, Philips Medical, Eindhoven, The Netherlands) using standard gradients and a circular polarized phase array head coil. For each subject, we acquired two series of functional volumes of T2^*^-weighted axial EPI-scans parallel to the AC/PC line with the following parameters: number of slices (NS), 22; slice thickness (ST), 5.0 mm; interslice gap (IG), 0.55 mm; matrix size (MS), 64 × 64; field of view (FOV), 240 × 240 mm; echo time (TE), 50 ms; repetition time (TR), 2.0 s. Four hundred and thirty-six functional volumes were acquired for the first part of the story and 270 functional volumes for the second part, adding up to 706 volumes in total.

### fMRI data analysis

MR images were analyzed using Statistical Parametric Mapping software (SPM5; www.fil.ion.ucl.ac.uk) implemented in MATLAB (v. R2006b, Mathworks Inc., Sherborn, MA). After discarding the first three volumes, all images were realigned to the first image to correct for head movement. Unwarping was used to correct for the interaction of susceptibility artifacts and head movement. After realignment and unwarping, the signal measured in each slice was shifted relative to the acquisition time of the middle slice using a sinc interpolation in time to correct for their different acquisition times. Volumes were then normalized into standard stereotaxic anatomical MNI-space by using the transformation matrix calculated from the first EPI-scan of each subject and the EPI-template. Afterwards, the normalized data with a resliced voxel size of 4 × 4 × 4 mm were smoothed with a 10 mm full width at half maximum (FWHM) isotropic Gaussian kernel to accommodate intersubject variation in brain anatomy. The time series data were filtered with a high-pass cut-off of 1/128 Hz. The autocorrelation of the data was estimated and corrected for.

Onsets for the simile phrases were set at the beginning of the phrase that referred to the explained element. The duration was measured individually for each simile and included the explained element, TOC, and explaining element. CS were modeled in a similar way, with the onset at the beginning of the phrase and the duration of the event being equal to the duration of the complete phrase. A random-effects group analysis was performed entering the contrast images for similes and CS from the first-level analysis into a full-factorial design matrix.

In a separate analysis, rating values for figurativeness, familiarity, and abstractness were entered individually as parametric variates for each simile into the first-level analysis in order to analyze correlations between brain responses during figurative speech processing and the aforementioned dimensions.

A further *post-hoc* analysis was performed with respect to the particular role of the TOC in the processing of abstract figurative speech. Therefore, the particular onset of the TOC (engl. “as” or “like”; e.g., in “He smokes like a chimney”) was individually calculated using an event-related design.

The results were corrected on a voxel-wise threshold of *p* < 0.001. Hereby, a Monte Carlo simulation of the brain volume of the current study was conducted to establish an appropriate voxel contiguity threshold (Slotnick et al., 2003). The procedure is based on the fact that the probability of observed clusters of activity due to voxel-wise Type I error (i.e., noise) decreases systematically as cluster size increases. Assuming an individual voxel type I error of *p* < 0.001 in our study, a cluster extent of 13 contiguous resampled voxels was indicated as necessary to correct for multiple voxel comparisons.

Each of the reported activations was determined with the Anatomy Toolbox for SPM5 (v. 1.7b, http://www.fz-juelich.de/inm/inm-1/spm_anatomy_toolbox). The imaging figures were made with the MRIcron software package (http://www.cabiatl.com/mricro/mricron/).

## Results

### Behavioral results

During the post-scan interview about critical story episodes, all participants were able to recall all the desired details in response to each of the 10 questions (Whitney et al., 2009b). Answers to all questions were provided quickly and effortlessly.

### fMRI results

#### Simile > CS

**Figure 1:**
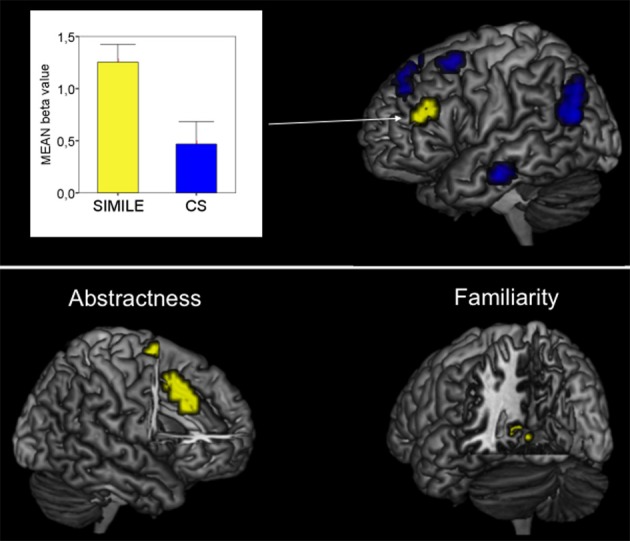
**Top:** Imaging results for the contrast Simile > CS. The bar graph illustrates the contrast estimates (beta values; yellow bar = Simile, blue bar = CS). **Bottom**: Imaging results for the interaction of brain responses with both familiar and abstract similes.

In the whole brain analysis, the simile sentences as contrasted with the CS selectively activated the left IFG including the pars triangularis and adjacent middle frontal gyrus (LMFG; *p* < 0.05, Monte Carlo corr.; Figure 1). Local maxima were found in both regions, though BOLD responses for the left IFG were stronger (Table 1). Extracted beta values for the activated region revealed activations for CS as well, however, activations were significantly stronger for the simile condition.

**Table 1:** **Peak activation within clusters for the contrasts Simile > CS, CS > Simile as well as for the correlation analysis with familiarity and abstractness (whole-brain analysis, Monte Carlo corr. *p* < 0.001)**.

		**BA**	**Coordinates**			***t*-value**	**No. voxels**
			***x***	***y***	***z***		
**SIMILE >CS**							
L	Inferior frontal gyrus (p. Triangularis)	45	−48	32	24	4.93	17
			−48	44	20	4.56	
**CS > SIMILE**							
R	Precuneus	7	4	−60	32	6.20	184
			0	−60	16	5.80	
			0	−44	36	4.85	
R	Middle temporal gyrus	20	52	−4	−24	6.12	44
			60	−12	−20	4.42	
L	Middle temporal gyrus	39	−56	−64	20	5.97	77
			−44	−60	24	5.54	
			−56	−68	32	5.12	
L	Middle frontal gyrus	6	−32	20	56	5.18	17
L	Middle temporal gyrus	20	−60	−12	−24	4.72	28
			−48	−16	−20	4.48	
R	Middle temporal gyrus	39	60	−64	12	4.69	102
			60	−60	24	4.64	
			52	−68	28	4.64	
**FAMILIARITY**							
L	Parahippocampal gyrus	30	−8	−40	0	4.50	25
**ABSTRACTNESS**							
L	Anterior cingulate	24	−4	24	28	4.87	42
			−8	16	36	4.47	
			0	8	44	3.46	
R	Superior frontal gyrus	6	12	4	72	4.01	14

Coordinates refer to MNI space.

#### CS > simile

A network of activations encompassing the precuneus, bilateral middle temporal gyri as well as the LMFG was found for the whole brain *CS* > simile contrast. A large cluster of activation extended from the left (BA 23) and right precuneus (BA 7) to left middle (BA 31) and posterior cingulate cortex (BA 7). Activations in the right middle temporal gyrus (BA 19) extended into the region of the angular gyrus (BA 39) as well as the middle occipital gyrus. Contralaterally, left middle temporal as well as the left angular gyrus (BA 39) were found to be more activated during CS processing. Enhanced BOLD responses were also found in the LMFG as well as in the bilateral middle and inferior temporal gyri (BA 20; Table 1).

### Correlation analyses

#### Familiarity

The correlation analysis between the degree of familiarity and BOLD signal changes revealed a pattern of activation in the left parahippocampal region (Table 1, Figure 1).

#### Abstractness

Highly abstract similes resulted in BOLD enhancements in the anterior cingulate cortex as well as in activations in the right superior frontal gyrus (Table 1, Figure 1).

#### Figurativeness

No significant relation was found between BOLD enhancements and figurativeness.

### *Post-hoc* analyses results

#### TOC > CS

Enhanced BOLD responses for the TOC as opposed to CS were found in the left-hemispheric IFG (p. triangularis) and the superior parietal region (Supplementary Material).

#### CS > TOC

The opposed contrast revealed pronounced activations in the right precuneus, middle temporal gyrus, and angular gyrus. In the left hemisphere, CS > TOC elicited neural responses in the middle temporal region (Supplementary Material).

#### ([TOC > CS] > [SIMILE > CS])

The contrast for TOC > CS as opposed to SIMILE > CS again resulted in left lateralized activations encompassing the IFG and the superior area of the parietal cortex (Supplementary Material).

#### ([SIMILE > CS] > [TOC > CS])

The inverse contrast resulted in right hemispheric activations in the precuneus and the middle temporal region (Supplementary Material).

#### SIMILE > CS ∩ TOC > CS

The conjunction analyses for both similes and TOC as contrasted with CS activated the left pars triangularis (Supplementary Material).

#### CS > SIMILE ∩ CS > TOC

CS as opposed to similes and TOC activated a neural pattern, encompassing the right precuneus, middle temporal gyrus as well as the angular gyrus. In the left hemisphere, enhanced BOLD responses were for the middle temporal region (Supplementary Material).

## Discussion

Figurative expressions, such as metaphors and similes are fundamental to language and thought. They represent a conventionalized part of everyday communication (Lakoff and Johnson, 2003). The processing of such figurative expressions requires a mental linkage between the explained and the explaining element. In case of a particular kind of metaphors, i.e., similes, this linkage is made explicit by the use of a TOC (“as” or “like,” German: “wie”), also referred to as a hedge word (Shibata et al., 2012). The aim of the present study was to investigate the neural basis of simile processing using a highly naturalistic, continuous speech perception paradigm. This allowed us to examine whether brain activation reported in previous investigations of figurative language in the left IFG also holds true in a natural setting of short story comprehension. In line with findings on metaphor processing (Rapp et al., 2004) we observed an involvement of the left IFG for the simile condition, suggesting that more neural resources are required to interpret the figurative meaning of the simile under naturalistic conditions. We moreover found significant correlations between enhanced BOLD responses in the anterior cingulate region and the superior frontal gyrus in the context of highly abstract figurative expressions. Correlation analysis for familiar similes resulted in activations in the left hippocampal region.

The neural processing of continuous, naturalistic stimuli has thus far only rarely been performed. Recent studies have either explored natural speech production (Kircher et al., 2000, 2004; Buchheim et al., 2006), narrative comprehension (Wilson et al., 2008; Whitney et al., 2009b; Brennan et al., 2012; Domahs et al., 2012), or naturalistic audio-visual processing mechanisms (Bartels and Zeki, 2004; Hasson et al., 2004, 2010).

### Simile processing in the left IFG

The results demonstrate that similes elicited enhanced BOLD responses in the dorsal part of the pars triangularis (left IFG) and the ventral portion of the LMFG when contrasted with non-figurative CS. The increased activation in these brain regions might reflect the enhanced demand on deep semantic processing integrating figurative expressions into the surrounding context. Based on the initial semantic conflict between explained and explaining element, the listener compares the figurative expression by means of a parallel, which is drawn to a different entity (“tertium comparationis”). Thus, common characteristics as well as distinct features of both elements are to be selected, emphasized, inhibited, and organized which goes beyond the usual level of contextual semantic word processing (Rapp et al., 2004).

Enhanced neural responses in the left IFG have previously been found in studies on metaphors compared to literal phrases using single sentences (Rapp et al., 2004). In a recent fMRI study, Schmidt and Seger (2009) compared sentences with easy and difficult metaphors. While easy metaphors were found to selectively activate the left MFG, difficult metaphors elicited enhanced BOLD responses in the left IFG. Similarly, the processing of metaphorical sentences taken from poetry resulted in activations of the left dorsolateral prefrontal cortex (Mashal et al., 2009). Bambini and colleagues investigated the neural correlates of implicit metaphor processing as compared to non-metaphorical passages, while being explicitly involved in an adjective matching task to be performed after reading the target passages (Bambini et al., 2011). The authors found a widespread neural network encompassing the left and right inferior frontal gyri, the right superior temporal gyrus, the left angular gyrus, and the anterior cingulate region. Imaging results were interpreted in terms of integrating linguistic material and world knowledge into the context. The left IFG and in particular the pars triangularis may hence represent a key region for figurative speech processing, including similes in a story context.

### Activation for control sentences

A widespread bilateral cortical network was found to be activated for control vs. simile sentences. Thus, enhanced BOLD responses were observed for CS in contrast to similes in the middle temporal gyrus bilaterally, the precuneus and the LMFG. Since the CS were matched with regard to plausibility and comprehensibility, enhanced activation may reflect general semantic analysis of concrete information continuously presented as previously found by Whitney et al. (2009a,b). The activations, in particular in the bilateral temporal gyri, were consistently reported for auditory language processing [for review see: Ferstl et al. (2008)]. Continuous listening to the concrete sentences includes inferences for bridging successive utterances, the use of background knowledge about concrete entities of the world and discourse context as well as lexical retrieval (Ferstl et al., 2008), all processes that have been attributed to the neural network found in our study.

The fact that the processing of similes resulted in a reduced involvement of this bilateral “concrete sentence” network indicates that either concrete representations were inhibited in favor of the relevant abstract interpretation, or the double representation (e.g., in the sentence “He smokes like a chimney!” the representation of smoke will be activated by both smoke and chimney) of the respective concept led to a facilitation of related processing mechanisms. Nevertheless, this finding in general supports the theory that abstract figurative meaning is mainly represented in the left hemisphere (Perlovsky and Ilin, 2010).

### Activations for familiarity

All of our similes were non-conventional, but more or less familiar to the listeners. We found a positive association between familiarity of similes and activation in the left parahippocampal region. No negative correlations with BOLD enhancements were found. These data suggest that familiar, lexicalized and therefore well-known similes, e.g., “serve like a slave,” are associated with enhanced semantic memory processes (Hoenig and Scheef, 2005). Thus, the enhanced semantic memory retrieval from the long-term storage as well as the integrative associative-mnemonic processes (Hoenig and Scheef, 2005) suggest a contribution of the left parahippocampal region to the processing of familiar figurative speech. With regard to the neural substrates of metaphor processing easy and familiar metaphors as contrasted with literal sentences have previously been found to activate the left parahippocampal gyrus (Schmidt and Seger, 2009). Similarly, Yang and colleagues (Yang et al., 2009) revealed activations in the bilateral hippocampal gyri for conventional metaphors, e.g., “She is a peach,” as opposed to a redundant condition such as “She is a female.” In the current investigation significant correlations for familiar similes were solely restricted to BOLD enhancements in the left parahippocampal region. A number of reasons may account for the selective recruitment of the parahippocampal gyrus. First, the linguistic structure of similes as compared to other metaphoric expressions differs with regard to the presence of a TOC (usually “like”). The presence of a mental linkage presumably facilitates the understanding of the figurative speech part, which might be explained by evoking wider associations of memory. Second, the auditory task design asking the participants to listen carefully to the narrative instead of reading or judging the isolated figurative or literal expressions, respectively, differs from recent experimental designs (Shibata et al., 2007; Schmidt and Seger, 2009; Yang et al., 2009).

### Activations for abstractness

In general, the anterior cingulate is involved in many processes, such as verbal working memory as well as in selective attention, online-monitoring processes, and abstract auditory sequencing (Carter et al., 1998; Macdonald et al., 2000; Lee et al., 2011). With regard to figurative speech processing, enhanced neural responses in the anterior cingulate were previously reported (Rapp et al., 2004; Shibata et al., 2007; Bambini et al., 2011; Diaz and Hogstrom, 2011). In the current study, however, BOLD enhancements in this region and in the right superior frontal gyrus were found for more abstract similes, such as “he moved forward like a swimmer against the tide.” This pattern of activation suggests an involvement of enhanced semantic selection and monitoring processes, since relevant aspects and appropriate literal meanings of the abstract explaining element must be filtered and interpreted. These enhanced cognitive control mechanisms together with the comparatively stronger abstract semantic integration demands may have resulted in the recruitment of the anterior cingulate.

### Activations for term of comparison

*Post-hoc* analyses for the TOC resulted in a neural pattern of activations encompassing left hemispheric pars triangularis as well as superior parts of the parietal region. The conjunction analyses with similes (including the whole figurative speech phrase) again resulted in BOLD enhancements in the pars triangularis. It might be hypothesized that the TOC early predicts the upcoming figurative information; the TOC represents a bridging element linking the concrete—the explained element—to the subsequent abstract mental image. The TOC (“like”) may support this linking process, since it explicitly points to the comparative nature of the utterance. Moreover, it can be assumed that the competition between the abstract and the literal meaning resulting in the additional recruitment of the left IFG (Chen et al., 2008).

### Limitations

Analyzing continuous and authentic speech perception in a natural context goes also along with a number of methodological problems. CS —though carefully matched—still represent an arbitrary selection that could differ in a specific aspect, which cannot be systematically controlled for. Finally, ratings (e.g., plausibility evaluations) have been performed on isolated sentences and consequently do not consider the specific narrative context. However, we could demonstrate a highly comparable result pattern for the processing of similes in contrast to CS previously found for highly controlled experiments on figurative speech processing (e.g., Rapp et al., 2004, 2007; Kircher et al., 2007). Correlation analyses moreover revealed plausible result patterns indicating that sentence evaluations are associated with corresponding cognitive mechanisms.

## Conclusions

The present study suggests that the left IFG plays a crucial role in the processing of figurative comparisons embedded into highly naturalistic continuous speech processing within a short story. These findings add novel plausibility to previous, highly restrained experiments and show the applicability of this approach. In general, future investigations may consider employing experimental paradigms, using ecologically valid and naturally evolving stimulus material, e.g., including contextual information or multi-modal processing (audio-visual perception), with a high resemblance to the real world.

### Conflict of interest statement

The authors declare that the research was conducted in the absence of any commercial or financial relationships that could be construed as a potential conflict of interest.
