# Supplementary material for: Neural substrates of figurative language during natural speech perception: an fMRI study
Source: Front Behav Neurosci. 2013 Sep 19;7:121. doi: 10.3389/fnbeh.2013.00121 (PMC3776934; doi:10.3389/fnbeh.2013.00121)
Supplement: Supplementary file 1 [file DataSheet1.DOCX]

Table 2: Peak activation within clusters for the contrasts TOC>CS, CS>TOC as well as for the contrast ([TOC>CS]>[SIMILE>CS]), [SIMILE>CS])>([TOC>CS], [SIMILE>CS])∩([TOC>CS] (whole-brain analysis, Monte Carlo corr., p<.05). Coordinates refer to MNI space.

|  |  |  | **Coordinates** | | |  |  |
| --- | --- | --- | --- | --- | --- | --- | --- |
|  |  | **BA** | **x** | **y** | **z** | **t-value** | **No. voxels** |
| **TOC>CS** | | | | | | | |
| L | Inferior Frontal Gyrus  (p. Triangularis) | 45 | -48 | 32 | 20 | 6.10 | 27 |
| L | Superior Parietal Lobe | 7 | -20 | -68 | 56 | 5.03 | 22 |
| L | Superior Parietal Lobe  (Intraparietal Sulcus) | 7 | -28 | -48 | 40 | 4.69 | 29 |
| **CS>TOC** | | | | | | | |
| R | Precuneus | 7 | 4 | -60 | 32 | 6.26 | 154 |
|  |  |  | 8 | -56 | 16 | 4.78 |  |
|  |  |  | -8 | -52 | 12 | 4.52 |  |
| R | Middle Temporal Gyrus | 20 | 52 | -4 | -24 | 6.12 | 39 |
|  |  |  | 60 | -12 | -20 | 4.78 |  |
| L | Middle Temporal Gyrus | 39 | -56 | -64 | 20 | 5.97 | 36 |
|  |  |  | -44 | -60 | 24 | 5.54 |  |
|  |  |  | -56 | -68 | 32 | 5.12 |  |
| R | Angular Gyrus | 39 | 48 | -72 | 40 | 5.21 | 123 |
|  |  |  | 48 | -60 | 16 | 4.97 |  |
|  |  |  | 56 | -48 | 12 | 4.80 |  |
| L | Middle Temporal Gyrus | 21 | -48 | -16 | -20 | 4.58 | 21 |
|  |  |  | -60 | -16 | -20 | 4.40 |  |
| **([TOC>CS]>[SIMILE>CS])** | | | | | | | |
| L | Inferior Frontal Gyrus  (p. Triangularis) | 45 | -48 | 32 | 20 | 6.35 | 31 |
| L | Superior Parietal Lobe | 7 | -20 | -68 | 56 | 5.01 | 27 |
|  |  |  | -20 | -52 | 56 | 3.94 |  |
| L | Superior Parietal Lobe  (Intraparietal Sulcus) | 7 | -28 | -40 | 40 | 4.90 | 30 |
| **([SIMILE>CS]>[TOC>CS])** | | | | | | | |
| R | Precuneus | 7 | 4 | -60 | 32 | 5.17 | 29 |
| R | Middle Temporal Gyrus | 21 | 52 | -4 | -24 | 5.03 | 21 |
|  |  |  | 60 | -12 | -20 | 4.14 |  |
|  |  |  | 64 | -8 | -12 | 3.45 |  |
| R | Middle Temporal Gyrus | 22 | 56 | -48 | 12 | 4.96 | 74 |
|  |  |  | 48 | -76 | 36 | 4.70 |  |
|  |  |  | 48 | -60 | 16 | 4.56 |  |
|  |  |  | 60 | -48 | 20 | 4.42 |  |
|  |  |  | 48 | -52 | 16 | 4.34 |  |
|  |  |  | 44 | -72 | 48 | 4.16 |  |
| **SIMILE>CS∩TOC>CS** | | | | | | | |
| L | Inferior Frontal Gyrus  (p. Triangularis) | 45 | -48 | 32 | 24 | 4.93 | 15 |
|  |  |  | -48 | 44 | 20 | 4.56 |  |
| **CS>TOC∩CS>Simile** | | | | | | | |
| R | Precuneus | 7 | 4 | -60 | 32 | 6.20 | 132 |
|  |  |  | 8 | -56 | 32 | 5.24 |  |
|  |  |  | 4 | -56 | 12 | 4.73 |  |
| R | Middle Temporal Gyrus | 20 | 52 | -4 | -24 | 6.12 | 32 |
|  |  |  | 60 | -12 | -20 | 4.42 |  |
| L | Middle Temporal Gyrus | 39 | -56 | -64 | 20 | 5.23 | 36 |
|  |  |  | -56 | -68 | 32 | 4.58 |  |
|  |  |  | -44 | -60 | 20 | 4.51 |  |
| R | Angular Gyrus | 39 | 52 | -64 | 24 | 4.58 | 83 |
|  |  |  | 48 | -72 | 40 | 4.52 |  |
|  |  |  | 64 | -52 | 24 | 4.41 |  |
| L | Middle Temporal Gyrus | 39 | -48 | -16 | -20 | 4.48 | 20 |
|  |  |  | -60 | -16 | -20 | 4.37 |  |
